# All atom NMDA receptor transmembrane domain model development and simulations in lipid bilayers and water

**DOI:** 10.1371/journal.pone.0177686

**Published:** 2017-06-05

**Authors:** Samaneh Mesbahi-Vasey, Lea Veras, Michael Yonkunas, Jon W. Johnson, Maria G. Kurnikova

**Affiliations:** 1 Department of Chemistry, Carnegie Mellon University, Pittsburgh, Pennsylvania, United States of America; 2 Department of Neuroscience and Center for Neuroscience, University of Pittsburgh, Pittsburgh, Pennsylvania, United States of America; Indiana University School of Medicine, UNITED STATES

## Abstract

*N*-methyl-d-aspartate receptors (NMDARs) are members of the ionotropic glutamate receptor family that mediate excitatory synaptic transmission in the central nervous system. The channels of NMDARs are permeable to Ca^2+^ but blocked by Mg^2+^, distinctive properties that underlie essential brain processes such as induction of synaptic plasticity. However, due to limited structural information about the NMDAR transmembrane ion channel forming domain, the mechanism of divalent cation permeation and block is understood poorly. In this paper we developed an atomistic model of the transmembrane domain (TMD) of NMDARs composed of GluN1 and GluN2A subunits (GluN1/2A receptors). The model was generated using (a) a homology model based on the structure of the NaK channel and a partially resolved structure of an AMPA receptor (AMPAR), and (b) a partially resolved X-ray structure of GluN1/2B NMDARs. Refinement and extensive Molecular Dynamics (MD) simulations of the NMDAR TMD model were performed in explicit lipid bilayer membrane and water. Targeted MD with simulated annealing was introduced to promote structure refinement. Putative positions of the Mg^2+^ and Ca^2+^ ions in the ion channel divalent cation binding site are proposed. Differences in the structural and dynamic behavior of the channel protein in the presence of Mg^2+^ or Ca^2+^ are analyzed. NMDAR protein conformational flexibility was similar with no ion bound to the divalent cation binding site and with Ca^2+^ bound, whereas Mg^2+^ binding reduced protein fluctuations. While bound at the binding site both ions retained their preferred ligand coordination numbers: 6 for Mg^2+^, and 7–8 for Ca^2+^. Four asparagine side chain oxygens, a back-bone oxygen, and a water molecule participated in binding a Mg^2+^ ion. The Ca^2+^ ion first coordination shell ligands typically included four to five side-chain oxygen atoms of the binding site asparagine residues, two water molecules and zero to two backbone oxygens of the GluN2B subunits. These results demonstrate the importance of high-resolution channel structures for elucidation of mechanisms of NMDAR permeation and block.

## Introduction

*N*-methyl-d-aspartate receptors (NMDARs) are glutamate- and glycine-gated ionotropic glutamate receptors (iGluRs) found mainly in postsynaptic membranes in the central nervous system [1, 2]. NMDARs are required for many forms of synaptic plasticity, including long-term potentiation and long-term depression, physiological processes that are involved in learning and memory. Dysfunction of NMDARs has been associated with multiple nervous system disorders such as schizophrenia, Alzheimer’s disease, bipolar disorder, post-traumatic stress disorder, cerebral ischemia-induced neuronal injury, and epilepsy [3–8]. Because of their broad involvement in brain function and disorders, NMDARs are targets of therapeutic interest [5, 9].

**Fig 1:**
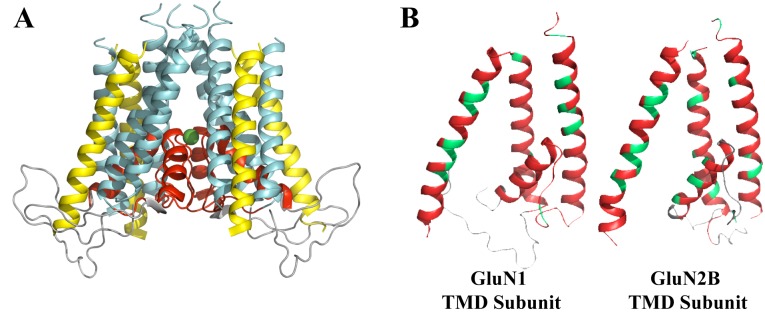
NMDAR transmembrane domain (TMD) homology model in relation to used templates. **A) The NMDAR TMD homology model color coded according to how the helices relate to the AMPAR [12] and NaK [13]templates.** A Mg^2+^ ion is shown in the core of the protein as the green sphere. Cyan corresponds to the regions that exist in the three structures and were well resolved in AMPAR and NaK channel structures, yellow corresponds to the regions that exist only in NMDAR and AMPAR and were well resolved in the AMPAR structure, red—to the regions that exist in the three structures and were well resolved in the NaK channel structure but not the AMPAR structure. The grey loops were not based in any template. **B) NMDAR TMD homology model color coded according to resolution in the NMDAR structure used as a template**. Residues are identified as unresolved (grey), partially (red) (only backbone and some side chain atoms were resolved) and fully (green) (all heavy atoms were resolved) resolved.

NMDARs are hetero-tetrameric protein complexes typically formed by two types of largely homologous subunits, GluN1 and GluN2. The physiological agonists for GluN1 subunits are glycine and d-serine, and for GluN2 subunits is glutamate. Functional tetrameric receptors contain two GluN1 subunits and two GluN2 subunits. There are four types of GluN2 subunits, GluN2A, GluN2B, GluN2C and GluN2D. Each GluN1 and GluN2 subunit is composed of an extracellular N-terminal domain (NTD), an agonist-binding domain (ABD), a transmembrane domain (TMD) and an intracellular C-terminal domain (CTD); see S1 Fig for the general structure and topology of NMDARs. The TMD tetramer, shown in Fig 1A, forms an ion-permeable pore, gating of which is controlled by the NTDs and ABDs. Each TMD monomer is composed of three transmembrane (TM) helices (M1, M3 and M4), and a pore loop composed of the M2 helix and an extended region. The extended region forms the narrowest part of the ion channel, called the selectivity filter. The selectivity filter structure plays a central role in ion permeation, ion selectivity, and channel block. NMDAR channels are permeable to monovalent cations including Na^+^ and K^+^, as well as Ca^2+^, and are blocked by Mg^2+^ in a membrane potential-dependent manner [6]. NMDAR channels can also be blocked by many organic compounds [6]. To a large extent the physiological importance of NMDARs is due to high selectivity of their ion channel for Ca^2+^. After entering a neuron through NMDARs, Ca^2+^ can initiate a cascade of biochemical events leading to important physiological processes including synaptic plasticity, or, in pathological conditions, to deleterious consequences including cell death [10, 11]. Voltage-dependent channel block by Mg^2+^ is responsible for controlling Ca^2+^ influx and plays a critical role in all aspects of NMDAR function.

In view of the physiological importance of NMDARs, NMDAR structure is a subject of active research [14]. The first two nearly full-length NMDAR structures of the GluN1/2B NMDAR subtype were partially resolved using X-ray crystallography [15, 16]. Modifications of the crystallized protein included truncation of its C-terminal domain and other smaller modifications, designed mostly to reduce protein flexibility. The overall resolution of the structures was a modest 3.7–4 Å resolution, although resolution varied significantly between domains of the protein. These two structures are of moderately high resolution within the extracellular NTD and ABD. However, the TMDs are of relatively low resolution and in parts unresolved [15, 16] (see Fig 1B). In particular, the extended region of the pore loop is missing from the structure published by Karakas et al. [15], and is partially resolved for only one of the subunits (subunit D) in the structure published by Lee et al. [16]. Recently, structures of lower resolution, which varied from 4 Å to 14 Å for different domains of the protein, were resolved using cryo-EM and X-ray crystallography [17–19]. These two structures also are low resolution in the TMD and are unresolved in the extended region of the pore loop. Therefore, critical structural information is missing for the region of the NMDAR ion channel that is specifically responsible for ion recognition, discrimination, and permeation, as well as for interaction with multiple pore blocking drugs.

Development of high resolution structural models for the TMDs of NMDARs is an essential step towards characterizing structural determinants that govern crucial properties of the ion channels. In recent years tools of the trade for theoretical prediction and refinement of protein structures have enjoyed rapid and impressive advances [20–25] also accelerated by rapid progress in computer technologies and novel programming techniques [26, 27]. While predicting structure of a large multi-domain complex such as an NMDAR ab initio is still out of reach for computational chemist, it is becoming increasingly possible to predict structures of smaller proteins and peptides from scratch [28, 29] as well as to model larger structures based on similarity to homologous structures. In this work we employ a combination of methods of protein modeling based on homologous protein templates [30], ab initio prediction of small protein structures and peptide loop structures [31] via minimization of energy functions, and molecular dynamics (MD) simulations [32] to develop a high resolution all atoms model of the NMDAR TMD. The homologous proteins used as templates for modeling NMDARs are structures of an AMPAR [12], a closely related and highly homologous iGluR, and of the NaK channel [13]. The NaK channel is a tetrameric non-selective cation channel closely related to potassium channels, which share a moderate degree of sequence similarity but highly similar secondary, tertiary, and quaternary structure with iGluR TMDs [33–35]. Use of two homologous structures to model NMDARs was important because in AMPAR structures, some of the TMD, in particular the extended region of the pore loop, were not fully resolved. Pore loops were well-resolved, however, in a number of structures of the more distantly related tetrameric channels, including the NaK channel.

The NMDAR TMD model presented in this study was built by homology modeling, then equilibrated and refined using molecular dynamics (MD) simulations, and includes structural information from crystallographic NMDAR structure [16]. The NMDAR TMD homology model was elaborated from an experimentally validated partial model of the NMDAR TMD [36] that used the NaK channel structure [13] as a template. Extensive further model refinements were performed using a combination of targeted MD simulations [37] with simulated annealing [38]. We demonstrate that the NMDAR TMD model is stable in simulations and has a well-formed divalent cation binding site that coordinates both Ca^2+^ and Mg^2+^. The residues that are believed to interact closely with Mg^2+^ and Ca^2+^ are six asparagines (Asn) that form a divalent cation binding site at the tip of the pore loop [39, 40]. Notably, the NMDAR TMD homology model presented in this study was fully developed, and subjected to MD simulations in lipid and water, prior to publication of crystallographically derived NMDAR structures. Therefore, by comparing our NMDAR TMD homology model and published crystal structures we are able to report an impressive success of template-based modeling techniques for this class of proteins. Specifically, the homology-based model is not only stable in the simulations but discrepancy between the homology model and the resolved parts of the crystal structures are less that uncertainty in the crystal structure itself, therefore the homology derived models are of similar resolution as published X-ray structures while it is an all atom model.

This paper is organized as follows. In Materials and Methods subsection A we describe development of the NMDAR TMD homology model (called stage 1 and stage 2 models), including incorporation of an Mg^2+^ ion in the predicted divalent cation binding site in the channel. In Materials and Methods subsection B we describe development of the MD-optimized NMDAR TMD model (stage 3 model) and the crystal structure-targeted NMDAR TMD model (stage 4 model). In Materials and Methods subsection C we describe simulation methodologies used in all reported MD simulations. In the Results section we describe MD simulations performed with the developed TMD model structures. In the Discussion and Conclusions section we present analysis of the NMDAR TMD properties deduced from our modeling and simulations.

## Materials and methods

### A) Template–based model development and incorporation of Mg^2+^ into a divalent cation binding site

**Fig 2:**
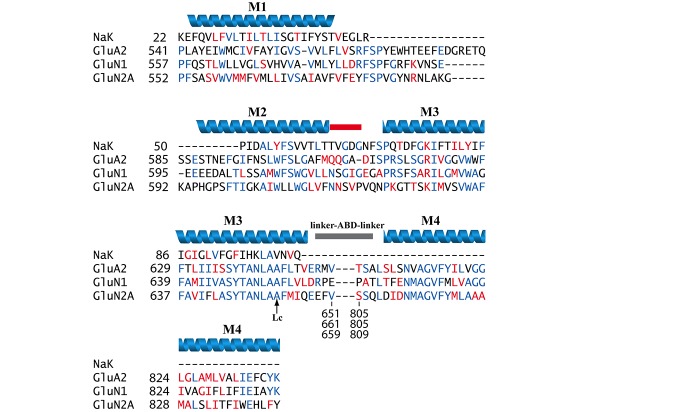
Sequence alignment of NaK channel, AMPAR (GluA2 subunit), and NMDAR (GluN1 and GluN2A subunits) TMDs. Identical residues are shown in blue and similar but not identical residues are shown in red. M1, M2, M3, and M4 as determined by MODELLER and inspection of the published crystal structures used here are shown in a blue ribbon representation. The non-helical part of M2 is marked as a red bar. The position of the Lurcher mutation is marked with an arrow labeled “Lc”. The M3-ABD linker, ABD, and ABD-M4 linker residues, which connect the M3 and M4 regions in iGluRs but are not found in NaK channels, are not shown in the figure.

The NMDAR TMD model developed in this work was built from a homology model of NMDARs using NaK and AMPAR crystal structures as templates. The model has been built in stages that are further referred to by stage numbers to simplify the description of a model-building protocol. Currently published NMDAR and AMPAR structures remain relatively unresolved in the core region of the channel, most importantly the M2 region. We therefore based the NMDAR channel core structure on the NaK channel [13]. The NaK channel is structurally similar to potassium channels [13], but, like NMDARs, is permeable to Ca^2+^ and Na^+^ as well as K^+^ [41]. Importantly, there is extensive evidence that potassium channels, such as KcsA and Shaker, share channel structure and geometry with iGluRs [33–35]. The NaK channel has been solved to high resolution [13, 42] and has been successfully used as a template for a homology model of the NMDAR M2-M3 regions [36]. Thus our stage 1 model for the M1, M2, and M3 regions of the NMDAR was developed based on published NaK channel crystal structures [13]. Because NaK channels do not have a transmembrane region homologous to the M4 of iGluRs, the AMPAR structure [12] was used to model the M4 region for our stage 2 model (Fig 1A shows the relation between the NaK channel and AMPAR structures and the NMDAR TMD homology model; Fig 2 shows sequence alignments).

#### Stage 1: NMDAR M1-M2-M3 homology model development

The sequences of *Rattus norvegicus* NMDAR subunits GluN2A (Uniprot entry code: Q00959; [43]) and GluN1 (UniProt entry code: P35439) [44] in the M1, M2, and M3 regions were hand aligned with the *Bacillus cereus* NaK channel sequence (UniProt entry code: Q81HW2) in accord with previously published alignments [34–36, 45]. The 2.4 Å resolution closed NaK channel crystal structure (PDB ID: 2AHY) [13] served as the structural template during homology modeling, which was performed using the MODELLER suite of programs [30]. An initial structure of the M1-M2-M3 portion of the NMDAR TMD was based on the NaK channel structure, except that the amino-acid residues that constitute the divalent cation binding site [2, 46] were remodeled as described below.

To optimize the NMDAR M1-M2-M3 homology model structure in the region of the selectivity filter we created a Mg^2+^ binding site using the side chains of the six M2-region Asn residues: N614 and N615 on each GluN2A subunit, and N616 on each GluN1 subunit [39, 40]. We determined the amide oxygen geometry that optimally coordinates Mg^2+^ using the Hartree-Fock ab initio method with 6-31G basis set as implemented in the HARLEM program [47]. Formamide molecules were used in lieu of Asn side chain models. The calculated distances between Asn amide-oxygens for each subunit are reported in Table 1 using the numbering scheme for NMDAR GluN1 and GluN2A. These distances were then used to introduce structural restraints while building the NMDAR M1-M2-M3 homology model in the presence of a Mg^2+^ atom. Specifically, distances between the Mg^2+^ ion and the oxygens in the formamide—Mg^2+^ complex were used as equilibrium distances of harmonic restraints (with a force constant of 0.5 kcal mol^-1^Å^-2^) between Mg^2+^ and the side chain oxygens of the six Asn residues during optimization of the protein structure in MODELLER.

**Table 1:** Quantum mechanics-derived distances for asparagine side-chain amide-oxygen coordination of Mg^2+^.

	GluN1 Site N	GluN2ASite N	GluN2A Site N+1	GluN1 Site N	GluN2A Site N	GluN2A Site N+1
**GluN1 Site N**	-	2.98	2.98	4.20	2.96	2.96
GluN2 Site N		-	2.98	2.96	2.96	2.98
GluN2 Site N+1			-	2.96	2.98	2.96
**GluN1 Site N**				-	2.98	2.98
GluN2 Site N					-	2.98
GluN2 Site N+1						-

The NMDAR subunits are identified as GluN1 and GluN2A. Each distance in the table, reported in units of angstrom (Å), corresponds to the calculated distance between one asparagine amide-oxygen atom and the asparagine amide-oxygen atom on another subunit. The distance between each asparagine oxygen reported in the table and the Mg^2+^ atom were all calculated to be 2.10 Å.

The Swiss-model program [48] was used to evaluate 100 models produced in MODELLER and identify 10 best models based on their Discrete Optimized Protein Energy (DOPE) scores. The loops between helices for all 10 models were modeled using the ROSETTA loop modeling application [31]. The most favorable (lowest energy) protein model was then solvated in water and a short equilibration MD simulation was performed to equilibrate positions of solvable loops. All MD simulations were carried out using the molecular dynamics software package Amber12 [49] and ff12SB force field [50] and TIP3P water. A typical simulation protocol is described in subsection C of Materials and Methods. In brief, equilibration of the loops was carried out at 300 K under NVT conditions (constant number of particles, constant volume and temperature) with an integration step of 2 fs. Temperature was maintained using the Langevine thermostat [51]. Hydrogen bonds were constrained via the SHAKE algorithm [52].

**Fig 3:**
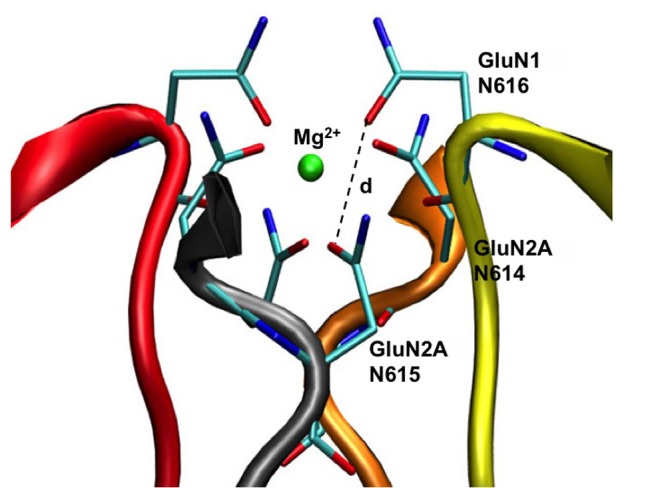
NMDAR asparagine carboxamide oxygen coordination of the Mg^2+^ ion. Each subunit secondary structure is shown in ribbon representation where subunit A is a GluN1 subunit colored yellow, subunit B is a GluN2A subunit colored orange, subunit C is a GluN1 subunit colored red, and subunit D is a GluN2A subunit colored grey. Three asparagine residues (N616 in GluN1; N614 and N615 in GluN2A) are shown in stick representations using a CPK coloring scheme. The single Mg^2+^ ion, which is shown as a green Van der Waals sphere, is coordinated by six amide-oxygens. Dashed line shows example of distance measurement between asparagine amide-oxygens (see Table 1).

The resulting structure of the divalent cation binding site with a coordinated Mg^2+^ and the adjacent loops of the M2 region is shown in Fig 3.

#### Stage 2: NMDAR TMD homology model development

We next used the crystal structure of a homomeric AMPAR composed of four GluA2 subunits (PDB ID: 3KG2) [12] to add the M4 region to the NMDAR M1-M2-M3 homology model. The protein structures from the NMDAR M1-M2-M3 homology model and the AMPAR crystal structure first were aligned by optimizing overlap of the C_α_ carbons coordinates of their M1 and M3 transmembrane regions. The M1 and M3 of the GluA2 subunits A and C were overlapped with the corresponding regions of the GluN1 subunits and the M1 and M3 GluA2 subunits B and D were overlapped with the GluN2A subunits. After overlapping the AMPAR structure and the NMDAR M1-M2-M3 homology model, the AMPAR M4 region was added to the NMDAR homology model. We then substituted each AMPAR M4 region residue with the corresponding GluN1 or GluN2A subunit residue in accord with the Sobolevsky et al. [12] sequence alignment. The resulting model was subjected to a brief energy minimization using Amber12 [49] to remove unfavorable interactions, producing the NMDAR TMD homology model. The root-mean-square deviation (RMSD) between the AMPAR crystal structure and the NMDAR TMD homology model computed for the M1 and M3 regions was 2.8 Å. The all-atom RMSD computed between M4s of GluN1 and GluA2 was 0.25 Å and between M4s of GluN2A and GluA2 was 0.20 Å.

To equilibrate the resultant NMDAR TMD homology model side chain packing, it was solvated in water, and a 1 ns MD simulation was performed under NVT conditions. In this simulation all C_α_ atoms were restrained using a harmonic potential with a large force constant (k = 20 kcal mol^-1^Å^-2^). [See the Materials and Methods subsection C for detailed description of the simulation protocols performed in this study].

### B) Molecular dynamics simulations performed for model optimization

#### Stage 3: MD-optimized NMDAR TMD model development

**Fig 4:**
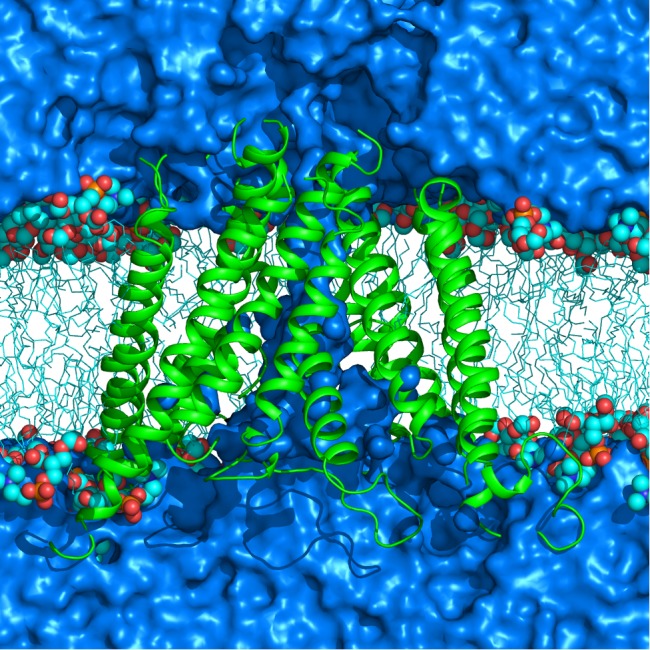
Simulated NMDAR TMD in DMPC lipid bilayer and water. The protein is shown as cartoon (green), lipid molecule hydrophobic tails as sticks (cyan) and lipid head-groups as spheres with carbon atoms colored in cyan, oxygen in red and phosphorus in orange. Water is represented by blue continuous medium. Water and lipids are truncated in the page plane to expose the channel.

The protein structure of the slightly equilibrated NMDAR TMD homology model was placed in a well equilibrated DMPC bilayer (Fig 4 shows a representative system of the TMD in lipid and water after equilibration and simulations as described below). DMPC parameters were taken from GROMACS [53]. The resulting system was solvated by water and extra water molecules were added to the TMD pore to ensure its adequate solvation. The final full system had 10159 water molecules, 108 DMPC membrane lipid molecules, and 534 protein residues, resulting in a total of 43850 atoms. The system was subjected to 3x10^5^ steps of energy minimization and a 1 ns isobaric-isothermic (NPT) ensemble MD simulation in which water molecules and the protein backbone were harmonically restrained with a force constant of 20 kcal mol^-1^Å^-2^ to allow lipid to equilibrate in the presence of the protein and water. The final size of the system was approximately 90 Å × 70 Å × 90 Å. Subsequent equilibrium MD simulations were performed as described in the Results section. A general protocol of MD simulations is described in subsection C of Methods section.

In the absence of ABDs the residues at the extracellular ends of M3 became water exposed, and the tips of the M3 helices were somewhat unstable during simulations. To help preserve helical structure of the M3 tips without imposing further restraints on the protein the following point mutations were introduced at the Lurcher position [54–59]: GluN1 A653Y and GluN2A A651Y. See the Discussion and Conclusions section for further consideration of the effect of these mutants on structural stability of the model system.

#### Stage 4: Crystal structure-targeted NMDAR TMD model development

These simulations are described in the Results section. The methods used in this stage of modeling are described in the following Materials and Methods Section C.

### C) General MD simulation methods

#### MD simulation protocols and parameters

All simulations in this work were carried out using the molecular dynamics software package Amber12 [49] using the pmemd.cuda program and ff12SB force field [50], TIP3P water model and DMPC lipid [53]. The MD simulations of the NMDAR TMD model in lipid membrane and water were performed with the integration time step of 1 fs. Pressure and temperature of the simulated system at equilibrium were maintained at 1 bar and 300 K respectively, employing the Langevin thermostat [60] with damping coefficient of 1 ps^–1^ and a semi-isotropic pressure scaling algorithm as implemented in Amber12 with pressure relaxation time of 5 ps. Hydrogen bonds were constrained via the SHAKE algorithm [52]. Periodic boundary conditions and all atom wrapping were employed in all simulations as implemented in AMBER. Non-bonded Lennard-Jones and Coulombic interactions were truncated at 12 Å. Long range electrostatics were calculated using the Particle Mesh Ewald method [61]. Images of molecular structures were created using PyMol software [62].

#### Targeted MD simulations and simulated annealing

Targeted MD is a method to facilitate transition of a simulated structure from its initial conformation to a desired target conformation [37]. In a targeted MD simulation a biasing harmonic potential (*U*) is applied to a subset of atoms (the target set) during a simulation. U is defined as:

$$U=\frac{1}{2}\frac{k}{N}[{RMSD(t)-{RMSD}^{*}(t)]}^{2},$$ (1)

where k is a force constant, *N* is the number of targeted atoms, RMSD (t) is an instantaneous Root Mean Square Deviation (RMSD) of the current coordinates to the target coordinates, and RMSD* (t) is a target RMSD value at time t.

In this study, targeted MD simulations were performed in order to incorporate NMDAR X-ray crystallography data [16] (PDB ID: 4TLM) into the MD-optimized NMDAR TMD model (stage 3 model). RMSD* (t) was set to 0. The external targeted MD force (the gradient of the potential *U*) was imposed on all C_α_ atoms resolved in the crystal structure [16]. Further details of the targeted MD application to refine the structure of the NMDAR TMD are described in the Results section.

Finding the ground state conformation of biomolecules is challenging due to the ruggedness of the free energy landscape, which is characterized by a vast number of local minima separated by a broad distribution of barrier heights. Simulated annealing is a central algorithm to find the global minimum of an empirical potential energy function (like the force fields used in MD simulations) [38]. In this work, employing simulated annealing allowed us to find local energy minima and therefore to better position the side chains. To perform simulated annealing, the external targeted MD force was applied while the system was heated and cooled. Therefore, in this study temperature is set to change throughout targeted MD simulations (where k is changing through Eq 1; see Results). The simulated annealing protocol generally consisted of equilibrating the NMDAR TMD model at 300 K for 1–6 ns, then raising the temperature to 350 K over a 1–3 ns period, then running the MD simulations at 350 K for 1–9 ns, and then decreasing the temperature back to 300 K over a 1–3 ns period.

The pmemd.cuda MD code in Amber 12 has been developed and optimized to perform calculations on a GPU (Graphic Processing Unit) which speeds up simulations by about three orders of magnitude compared to running the pmemd module on CPUs (Central Processing Units). However targeted MD protocol in Amber12 has been implemented only in the pmemd module. Therefore, in order to speed up our calculations, we redefined the targeted MD protocol in Amber12 to be able to perform simulations using the pmemd.cuda version.

## Results

### NMDAR TMD homology model refinement using MD simulations in membrane and water

**Fig 5:**
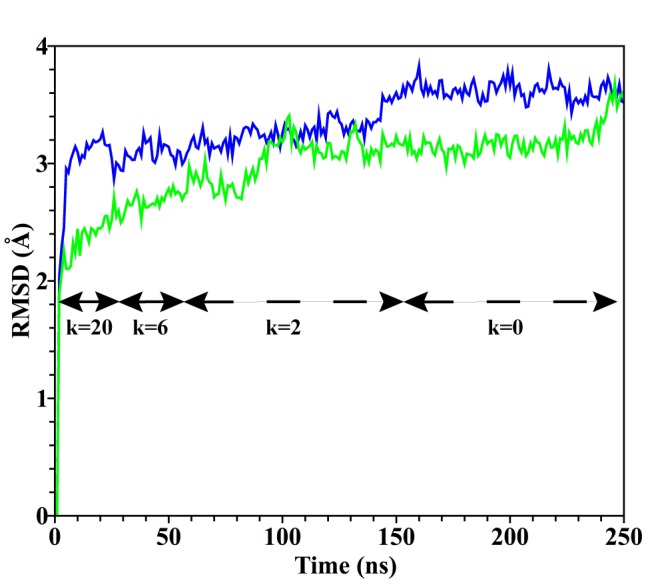
RMSD of all helical C_α_s during 250 ns of MD simulations performed during development of the MD-optimized NMDAR TMD model. RMSD was calculated as the deviation from the starting structure, the NMDAR TMD homology model. Results for the simulation with Mg^2+^ in the divalent cation binding site are shown in green, and for the simulation with Ca^2+^ in blue. *k* is the force constant in kcal mol^−1^Å^−2^.

In this section we present results of MD simulations used to develop, refine and simulate the MD-optimized NMDAR TMD model (stage 3) in membrane and water. Fig 4 shows an image of a representative simulated structure of the protein embedded in lipid bilayer and water, with Mg^2+^ bound in the channel. Using NMDAR TMD homology model slightly refined in MD simulations (as described in Methods, model stage 3) we performed two independent MD simulations with either Mg^2+^ or Ca^2+^ bound in the divalent cation binding site. In these simulations the NMDAR TMD homology model was initially equilibrated as follows: the hydrogen bonds and the dihedral angles of all TMD helices were harmonically restrained with a force constant of 20 kcal mol^-1^Å^-2^ for 25 ns. The strength of the restraints was gradually reduced to 6 kcal mol^-1^Å^-2^ for 25 ns, and then to 2 kcal mol^-1^Å^-2^ for subsequent 100 ns. Finally, all restraints were removed and the two systems (one with Mg^2+^ and the other with Ca^2+^ in the divalent cation binding site) were simulated for 100 ns. A total of 250 ns of isobaric-isothermic equilibrium simulations were performed with each of the systems. The final structure, the MD-optimized NMDAR TMD model, was stable with Mg^2+^ or with Ca^2+^ in the divalent cation binding site. During the 250 ns of simulations that started with the NMDAR TMD homology model, the protein structure deviated from its starting structure by 3.5 Å (computed average RMSD of all helical C_α_ atoms as a function of time, see Fig 5). There was little deviation of the equilibrated structure during the last 100 ns of the simulations in absence of all restraints on the protein. In both simulations a divalent ion remained bound in the binding site and did not deviate from its initial position. We also evaluated Root Mean Square Fluctuation (RMSF), which shows the motion of each residue with respect to its average position in equilibrium simulations (data not shown). We observed small RMSF values for helical core residues of each subunit during unrestrained equilibrium simulations (over the last 100 ns of each trajectory shown in Fig 5) in simulations with either Mg^2+^ or Ca^2+^ in the divalent cation binding site, which indicates a stable core structure for each of the subunits. As expected, the loops and the terminal amino acid residues of the protein exhibit greater average flexibility (not shown) than the helical residues. The stability of the secondary structure was also monitored by measuring helicity (data not shown). Helicity is the percentage of the residues in the helical regions that remained in helical conformation throughout the simulation. During the simulations, all α and 3–10 helices maintained a helicity greater than 90%.

### Refinement of the MD-optimized NMDAR TMD model by targeted molecular dynamics and simulated annealing

The NMDAR TMD model built by homology modeling and equilibrated in lipid and water (as described in the previous section) was subjected to Targeted MD simulations with simulated annealing to incorporate crystallographically determined structural information from Ref. [16]. As a starting structure for the targeted MD refinement simulations we chose the MD-optimized NMDAR TMD (stage 3) model with Mg^2+^ coordinated at the divalent cation binding site after 150 ns of the MD simulations described above. This structure was well equilibrated in membrane and water and had not significantly deviated from the starting homology model in extensive MD simulations (Fig 5). The stage 3 model structure after 150 ns of MD simulation had an RMSD to the Lee et al. [16] crystal structure (PDB ID: 4TLM) of 3.79 Å (S2 Fig), which is well within the resolution of the crystal structure in the TMD. The 4TLM structure was chosen as a target structure because in this structure more residues were resolved, and overall resolution was higher, than in the Karakas et al. (2014) structure [15]. The C_α_ RMSD between TMDs of the two NMDAR structures [15, 16] is 1.37 Å. All the TMD C_α_ carbons that were resolved in the crystal structure [16] were chosen as the target set for the targeted MD simulations. The Mg^2+^ ion was retained in the divalent cation binding site.

**Fig 6:**
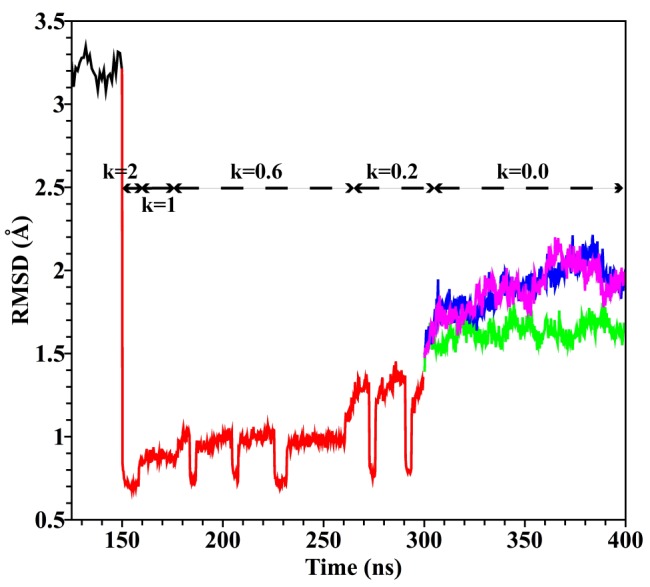
RMSD of the target set of C_α_ atoms in simulations of the crystal structure-targeted NMDAR TMD (stage 4) model. The RMSD was calculated with respect to the target NMDAR crystal structure. The black line shows part of the equilibrium MD trajectory of the homology model simulations also shown in Fig 5 (corresponding to 125–150 ns with Mg^2+^ at the divalent cation binding site in Fig 5). This simulation is shown to demonstrate the continuity of the model building and refinement procedures employed in this work; the red line corresponds to targeted MD with simulated annealing; see text for details. The k values in kcal mol^-1^Å^−2^ indicate force constant in targeted MD simulations. The green, blue and pink lines correspond to restraint-free simulations of the crystal structure-targeted NMDAR TMD model (stage 4) with Mg^2+^, Ca^2+^ and no ion in the binding site, respectively. Brief periods during the cooling phase of simulated annealing when k was raised from 0.6 or 0.2 to 2.0 kcal mol^-1^A^-2^ are not marked, but can be recognized by the drop in RMSD values.

A summary of all simulations described in this subsection that were used to develop the crystal structure-targeted NMDAR TMD model (stage 4) is shown in Fig 6. The figure shows the RMSD of all targeted C_α_s with respect to the NMDAR crystal structure. For a general description of methods see Materials and Methods section C.

In detail, the targeted MD with simulated annealing protocols employed were as follows (see Fig 6 for the scheme and results of the simulations described here). The MD-optimized NMDAR TMD model was targeted to the NMDAR crystal structure using a force constant of 2 kcal mol^−1^Å^−2^. Two rounds of simulated annealing were performed resulting in total of 8.25 ns of MD simulations during this stage. The strength of the targeting restraints was then reduced to 1 kcal mol^−1^Å^−2^ and three more rounds of the annealing were performed. A total of 19.5 ns of MD simulation were completed during this stage. Then, three rounds of further annealing were performed, with the targeting force constants as follows, the force constant was reduced to 0.6 kcal mol^−1^Å^−2^ during heating and increased to 2.0 kcal mol^−1^Å^−2^ during cooling, respectively. This ensured the backbone atoms approach the target positions during cooling simulations. A total of 83 ns of targeted MD simulations were completed during this stage. The force constant of the targeting restraint was then decreased to 0.2 kcal mol^−1^Å^−2^ and two rounds of annealing were performed, with k raised to 2.0 kcal mol^−1^Å^−2^ during cooling. A total of 39 ns of MD simulation were completed during this stage.

After completing the targeted MD with simulated annealing simulations the equilibrated structure of the protein was used to perform equilibrium MD simulations in the absence of any restraints. Three independent unrestrained simulations were performed for 100 ns each: one with the continued presence of Mg^2+^ at the divalent cation binding site (Fig 6, green line), one with Mg^2+^ replaced by Ca^2+^ (Fig 6, blue line) (accomplished by changing the Lennard-Jones parameters from Mg^2+^ values to Ca^2+^ values; Fig 6, blue line), and one with no ion at the cation binding site (Fig 6, pink line). In addition, a simulation, in which the first ligand coordination shell of the Mg^2+^ ion was restrained to include the six carboxamide groups of the asparagines that comprise the divalent binding site (as in Fig 3) was performed to equilibrate the symmetric Mg^2+^ coordination sphere. After 30 ns all restrains on the ion and the ligands were removed and the structure was simulated at equilibrium. No ligand exchange near Mg^2+^ ion was observed in any of the simulations that included Mg^2+^ ion.

Targeted MD with annealing simulations resulted by design in an NMDAR TMD model structure that corresponded closely to the NMDAR crystal structure (see Fig 6; RMSD at 300 ns = 1.3 Å). In the subsequent 100 ns of unrestrained simulations (corresponding to 300–400 ns in Fig 6), the target set C_α_s RMSD in all three independent simulations increased initially by about 1.5 Å and reached values of 2.3 Å after 70 ns of simulation for the Ca^2+^ and no ion cases. However in the Mg^2+^ case, the total C_α_s RMSD increased only to 1.7 Å. The protein structure became and remained stable in all three simulations with only small fluctuations for the final 30 ns of the simulations. The time average RMSDs per residue (calculated for the target set of C_α_s with respect to the crystal structure) for the last 100 ns are shown in S3 Fig.

**Fig 7:**
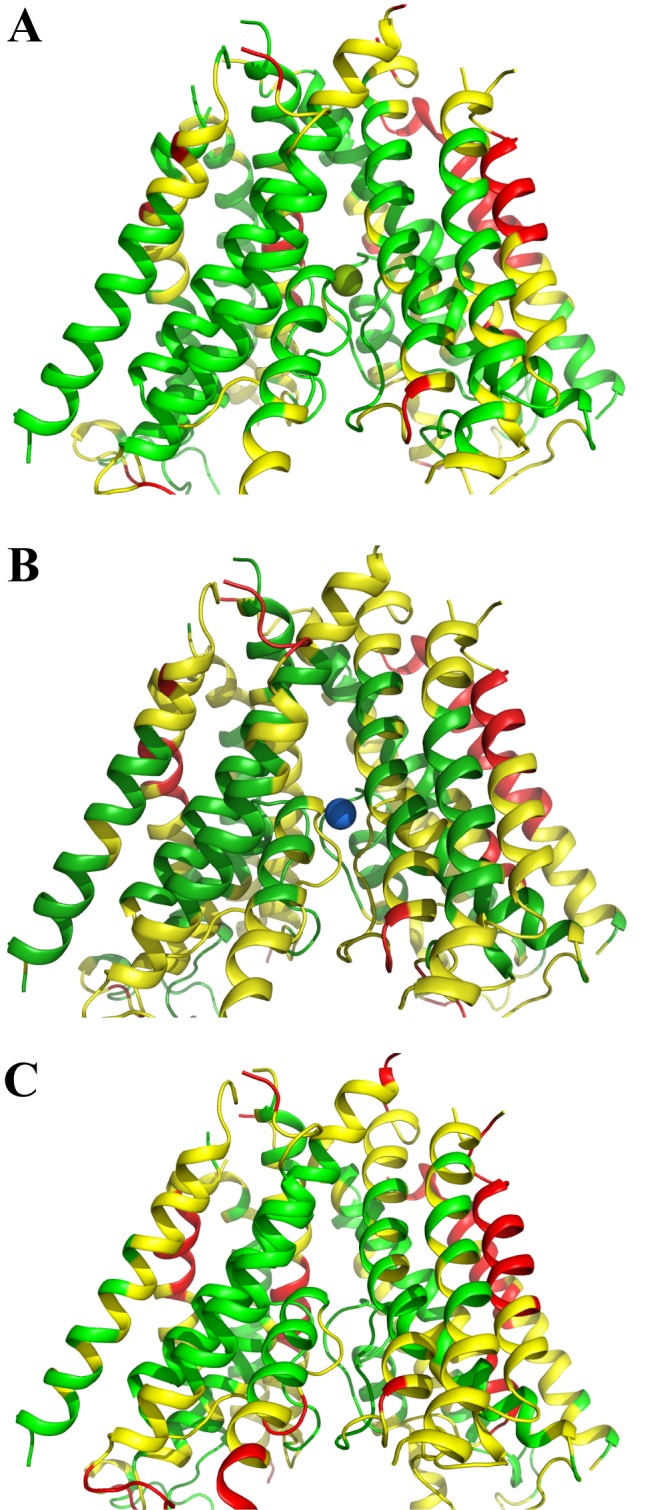
Crystal structure-targeted NMDAR TMD (stage 4) model color coded according to RMSF per residue. RMSF values were calculated for the last 50 ns of restraint free simulation (Fig 6) with the divalent cation binding site occupied by Mg^2+^ (A), Ca^2+^ (B), or no ion (C). RMSF values are color-coded as follows: green, less than 0.7 Å; yellow, 0.7 to 1.5 Å; red, more than 1.5 Å.

The RMSDs of individual helices (calculated for C_α_s and with respect to the crystal structure) are shown in S4 Fig. Structures of the crystal structure-targeted NMDAR TMD model at the end of the three simulations (at the 400 ns time point in Fig 6) are shown in Fig 7.

## Discussion and conclusions

We have used homology modeling and extensive MD simulations, including a novel combination of targeted MD with simulated annealing, to develop and refine a high-resolution all atom structural model of the GluN1/2A NMDAR transmembrane domain. A total of one microsecond of MD simulations of the NMDAR TMD structures embedded in lipid bilayer and water were performed. In the first set of the MD simulations described in this work (Fig 5), which started with the NMDAR TMD homology model, a total of 500 ns of MD simulations were performed with either Mg^2+^ or Ca^2+^ in the divalent cation binding site. In the second set of the simulations (Fig 6), which started from the MD-optimized NMDAR TMD model, 450 ns more of simulations that incorporated NMDAR X-ray crystallographic information were performed. During the last 100 ns of the second set of simulations, Mg^2+^, Ca^2+^, or no ion was present in the divalent cation binding site.

The final crystal structure-targeted NMDAR TMD (stage 4) model is within 2 Å (RMSD of resolved C_α_ atoms) of a published NMDAR crystal structure [16]. The RMSD and RMSF values for the helical parts of the protein indicate that the structure of each subunit is very stable in the simulations with Mg^2+^, Ca^2+^, or with no ion bound to the divalent cation site in the channel (see Figs 6 and 7 and S3 and S4 Figs). The loops and the terminal residues of the protein model exhibit greater average flexibility compared to the helical parts, as expected. The channel is continuously hydrated up to the helix bundle crossing forming the narrowest part of the channel, which is lined by the non-polar residues of the conserved SYTANLAAF motif (see Fig 4). In all simulations the channel remained in closed conformation.

In our MD simulations the tops of the M3 helices were truncated just above the hydrophobic channel constriction because the M3-ABD linkers and ABDs were not included in our NMDAR TMD models. Thus, the tops of the M3 helices, which interact with the M3-ABD linkers in the full-length receptor, instead were exposed to water and to the hydrophilic lipid head-group regions. As a result, the tops of the M3 helices lost helicity in simulations due to the exposure of their hydrophobic residues to water and lipid head groups. Introduction of hydrophilic residues at the Lurcher positions [54–59] in our MD simulations was found to help the M3 helix retain its helical structure in this region in the absence of the M3-ABD linkers and ABDs. The Lurcher position mutations should not have had long-range effects on TMD dynamics or structural stability because the simulations were too brief for the protein to explore conformational states beyond the modeled (closed channel) state. In functional full length NMDARs, Lurcher position mutations can increase open probability of the channel, and thus partially decouple the ABD from the TMD.

An inter-subunit pair of residues, GluN2A(S632) and GluN1(W608), that are expected to be in close proximity based on a mutant cycle analysis [36], were indeed separated by only about 8 Å in our stage 3 and 4 models with Ca^2+^ bound at the divalent cation binding site (S5 Fig). In both published X-ray structures [15, 16] the side chain of GluN2A(S632) was not resolved and the residue was modeled as alanine. In Karakas et al. [15] the side chain of GluN1(W608) was not resolved and it was modeled as alanine; in Lee et al. [16] the side chain of GluN1(W608) was resolved. The neighboring tryptophan residue, W611, was resolved as Ala in both published structures. In the Lee *at al*. [16] structure the distances between GluN2A(S632) and GluN1(W608) residues were between 8.2 Å and 9.02 Å, and in the Karakas et al. [15] structure these distances were between 7.01 Å and 7.91 Å, which is consistent with our models with Ca^2+^ bound. In simulations with Mg^2+^ at the binding site these residues were on average separated by only 4.5 Å, suggesting that divalent cation binding site occupation by Mg^2+^ brings these two residues closer together. It is important to note that the distances separating the equivalent residues in the AMPAR crystal structure [12] were approximately 20 Å or greater, supporting the choice of the NaK channel as a better template for modeling the NMDAR M2-M3 regions.

**Fig 8:**
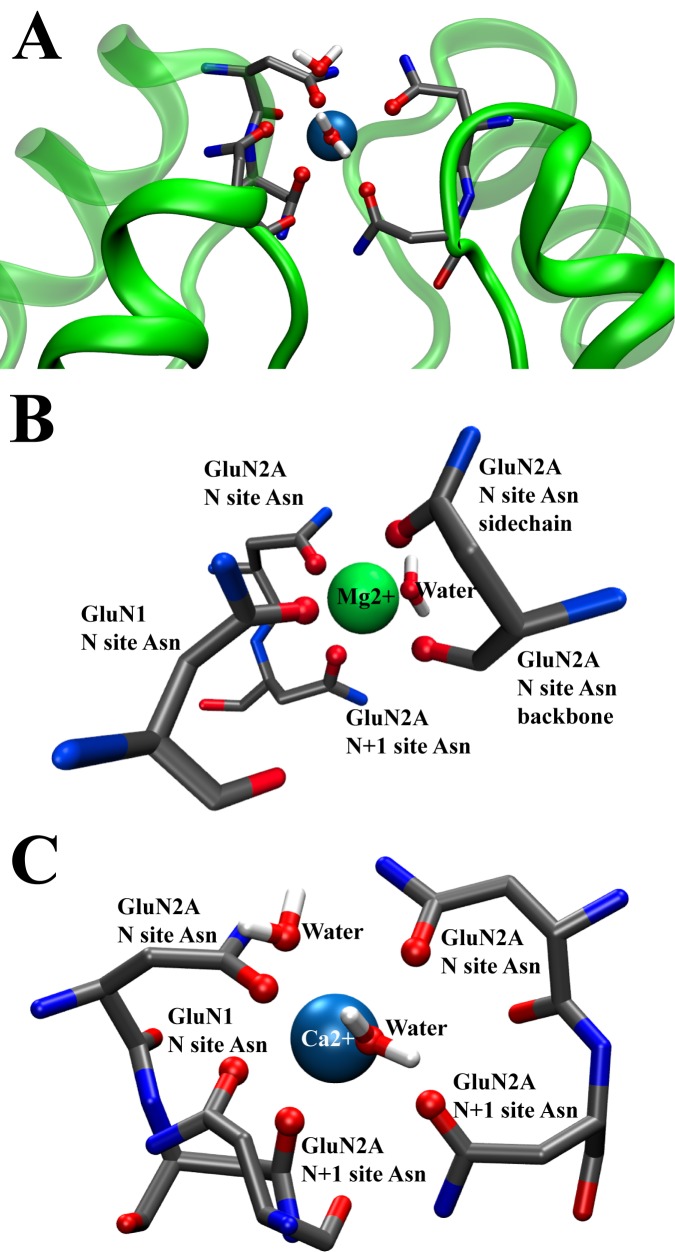
Divalent ion coordination at the tip of selectivity filter A) Ca^2+^ (blue sphere) at the tip of the selectivity filter coordinated by asparagine side chain oxygens and water. B) Mg^2+^ (green sphere) coordinated by six ligands: four asparagine side chain oxygens, one asparagine backbone oxygen, and one water molecule. C) Ca^2+^ coordinated with seven ligands: five asparagine side chain oxygens and two water molecules. Coordinating oxygens are shown as red spheres in panels A-C. The snapshots are taken from the restraint-free equilibrium MD simulations (corresponding to 300–400 ns in Fig 6).

One important goal of this work was to develop a structural model of the ion pore region and the divalent cation binding site located within the NMDAR TMD. Our simulations help resolve an important question: how can the NMDAR channel distinguish Ca^2+^ and Mg^2+^ so exquisitely, despite the chemical similarity of two ions. While Mg^2+^ has a smaller diameter than Ca^2+^ it can block NMDAR channel and it may permeate only in the presence of extreme voltage [63, 64] or absence of external permeant ions [65]. Ca^2+^, which is significantly larger in size, is the preferred permeant ion. Our simulations shed light on the differences between Ca^2+^ and Mg^2+^ interactions with the protein groups forming the divalent cation binding site. In our model, the six M2 region Asn residues line the pore at the tip of the selectivity filter with their side chains extended into the water-filled central cavity of the pore (Figs 3 and 8A). These six asparagines were previously implicated in divalent ion recognition by NMDAR channels [39, 66, 67], albeit their roles in channel permeation and blocking differ [68]. The carboxamide groups of the Asn side chains are solvated by water in the central cavity of the channel, and may form hydrogen bonds with each other. They can also compete with water for a position in the first coordination shell of an incoming divalent cation thus capturing the ion solvated in water in the wide region of the pore. The process by which NMDAR channels capture an ion therefore starts with partial desolvation of the ion as it enters the channel constriction formed by the M2 pore loops and interacts with pore-lining residues. Such substitution of ligand in the first solvation shell of an ion is plausible for Ca^2+^ and Mg^2+^ because of their preference for carbonyl and carboxamide ligands compared to water [69, 70]. Carbonyl or carboxamide containing agents are widely used, for example, to scavenge divalent ions from water solutions [71]. Here we show that the Asn side chains that form the divalent cation binding site can coordinate either divalent cation (Fig 8B and 8C) by forming a flexible cage, but that the structure and dynamics of the ion coordinating binding site cage is different for Ca^2+^ and Mg^2+^.

In solution, both Ca^2+^ and Mg^2+^ ions partition similarly between water and other oxygen-containing solvents. The main differences in ligand coordination for Ca^2+^ and Mg^2+^ are in the coordination numbers: Mg^2+^ requires six ligands, while Ca^2+^ may be coordinated by anywhere between 6 to 8 ligands; the rates of the first coordination shell ligand exchange, and the ligand exchange mechanisms, which is dissociative for Mg^2+^ and associative for Ca^2+^ [72, 73]. Briefly, in a dissociative ligand exchange mechanism, one ligand is lost from the first coordination shell before the new one enters it; in an associative mechanism, a new ligand joins the ion coordination shell before an extra one leaves it [72]. Therefore, a transient complex coordination number is 5 for Mg^2+^ and 7–9 for Ca^2+^. Our equilibrated model structure demonstrates that the six Asn side chain oxygens may form a complete Mg^2+^ coordination cage as shown in Fig 3. In the simulation in which a symmetric Mg^2+^ binding cage was strictly enforced using restraints during long equilibration simulations, the Mg^2+^ coordination cage remained stable in the subsequent unrestrained simulations (not shown). However, in simulations that used a standard equilibration procedure (Fig 5) the structure of the divalent cation binding site was persistently asymmetric (in several repeat simulations not shown here). A configuration that formed repeatedly in the simulations (Figs 5 and 6) had five Asn oxygen atoms (four provided by the Asn side chains and one provided by an Asn backbone oxygen), and one water in the Mg^2+^ first coordination shell (see Fig 8B). In all simulations the Mg^2+^ ion coordination number was six, as expected, throughout the MD simulations. There was no Mg2+—ligand exchange in the course of any of the equilibrium simulations, also as expected, due to the slow rate of Mg2+—ligand exchange [72, 73] and the relatively short duration of the simulations. Asymmetry of the Mg^2+^ binding site is consistent with the observation that in almost all Ca^2+^ and Mg^2+^ binding proteins with known structures, binding sites are formed by no more than four Asp (or Glu), or Asn side chains [74, 75]. The rest of the ion-coordinating oxygen atoms are provided by the protein backbone carbonyl groups and water [73, 76]. Therefore, the observation in our simulations that only four out of six Asn side chains simultaneously participate in coordinating Mg^2+^ appears consistent with typical structure of the divalent cation binding sites.

The behavior of the ion coordinating region of the NMDAR TMD was significantly different in simulations in which Ca^2+^ was placed in the divalent cation binding site. The total number of oxygen ligands coordinating Ca^2+^ varied from five to eight; in the majority of the simulations, Ca^2+^ was coordinated by seven oxygen ligands (Fig 8C). Moreover, the oxygen atoms in the Ca^2+^ first coordination shell (within 2.5 Å of an ion) exchanged during the simulations at a frequency of 5 × 10^10^ s^−1^. Ca^2+^ was coordinated mostly by Asn side-chain oxygens. In some of the complexes at most two backbone oxygens from the GluN2A subunit coordinated Ca^2+^ at the same time. Typically two water molecules were found in the first coordination shell of the Ca^2+^ ion. See S6 Fig for an illustration of Ca^2+^ first coordination shell occupancy during targeted MD simulations.

In this work we have not made an attempt to enumerate all possible coordination configurations for the Mg^2+^ ion, nor have we estimated their relative energies (that will be the subject of a future study). The difficulty of modeling Mg^2+^ ions in classical MD simulations is well known and stems mainly from its high density of charge and slow ligand exchange rate [72]. During the typical time span of ion diffusion and binding in an ion channel, multiple ion coordination configurations are likely to occur. Multiple low-energy ion-ligand configurations may be explored during channel occupancy by an ion, thus increasing the ion’s affinity for the channel by increasing entropy and lowering the free energy of ion-channel interaction. It is reasonable to expect that all relevant configurations of the bound Mg^2+^ will have six, or sometimes five, oxygen ligands. In the NMDAR TMD divalent cation binding site there are multiple possible configurations involving several Asn side chain oxygens, nearby backbone oxygens, and water.

Lastly, it is worth noting that the MD-optimized NMDAR TMD model (stage 3 model) was developed prior to publication of high resolution X-ray based NMDAR structures. Therefore, these models provided us with an opportunity to examine the performance of homology and MD simulation modeling methods for large protein systems. As was described in Results, the MD-optimized NMDAR TMD model was very stable and showed a well-formed divalent cation binding site for Ca^2+^ and Mg^2+^. When overlapped with all C_α_s resolved in the NMDAR crystal structure (S2 Fig), the measured RMSD of the MD-optimized NMDAR TMD model structure was 3.79 Å, in excellent agreement and on par with the overall crystal structure resolution in the TMD. GluN2A subunits show weaker homology to the NaK channel than the GluN1 subunits. Probably as a result, GluN2A subunits contributed more to the overall C_α_ RMSD from the initial stage 2 model during the MD simulations used to create the stage 3 model.

In conclusion, the development of fully atomistic NMDAR TMD models is presented in this work. Our models are stable during extensive MD simulations in explicit lipid bilayer and water. We will now be able to study the detailed mechanisms of divalent cation permeation and block of NMDARs. In this study the main results include observation of: lower protein flexibility when Mg^2+^ was bound in the divalent cation binding site than when Ca^2+^ was bound or when the site was unoccupied; ion-dependence of ion coordination numbers, with Mg^2+^ maintaining six oxygen coordination, but Ca^2+^ maintaining an average coordination number of 7 ligands (with a range of 6 to 8). Mg^2+^ was preferentially coordination by four Asn side chain carboxamide oxygens, one backbone Asn oxygen, and a single water molecule. Future simulations including *in silico* mutations of the selectivity filter Asn residues that are known to alter ion selectivity and blocking properties can help shed light on the mechanisms by which the NMDAR channel distinguishes Mg^2+^ and Ca^2+^.

## Supporting information

S1 Fig Domain organization of the GluN1/2B NMDAR. Domain organization of the NMDAR is shown for GluN1/2B subtype [16] (PDB ID: 4TLM). View of the receptor complex is parallel to the membrane, with the GluN1 subunits in cyan and yellow and the GluN2B subunits in green and purple.
(PNG)

S2 Fig Alignment of MD-optimized NMDAR TMD model with NMDAR crystal structure. Superposition of the MD-optimized NMDAR TMD model (beige) and the Lee et al. [16]crystal structure (cyan). The total RMSD for all C_α_s that were resolved in the crystal structure and included in the MD-optimized NMDAR TMD model is 3.79 Å. Panel A is side view and panel B is the top view (from the extracellular side) of the NMDAR.
(PNG)

S3 Fig Average RMSD per residue (calculated for M1, M2, M3, M4 C_α_s) of the last 100 ns (where all restraint were released) of targeted MD simulations. RMSDs were computed compared to the NMDAR crystal structure (calculated for C_α_s) for the last 100 ns (corresponding to 300–400 ns in Fig 6) of the targeted MD simulations (stage 4). Simulations with Mg^2+^, Ca^2+^ and no ion in the divalent cation binding site are shown in green, blue and pink, respectively. In each panel, A and C correspond to GluN1 subunits and B and D correspond to GluN2A subunits. The numbering scheme is as follows:
Panel A Residue numbers 2–25 and 65–88 correspond to GluN1 residues 558–581 in PDB ID: 4TLM. Residue numbers 27–44 and 46–63 correspond to GluN2A residues 551–568 in PDB ID: 4TLM.
Panel B Residue numbers 1–13 and 26–38 correspond to GluN1 residues 592–604 in PDB ID: 4TLM. Residue numbers 14–24 and 40–50 correspond to GluN2A residues 586–596 in PDB ID: 4TLM.
Panel C Residue numbers 1–26 and 82–107 correspond to GluN1 residues 617–642 in PDB ID: 4TLM. Residue numbers 28–53 and 55–80 correspond to GluN2A residues 611–636 in PDB ID: 4TLM.
Panel D Residue numbers 1–27 and 31–58 correspond to GluN1 residues 800–827 in PDB ID: 4TLM. Residue numbers 62–89, and 91–118 correspond to GluN2A residues residue 800–827 in PDB ID: 4TLM.
(PNG)

S4 Fig RMSD of individual helices (M1, M2, M3, M4) (calculated for C_α_s) of the last 100 ns (where all restraints were released) of targeted MD simulations (stage 4) (shown for Ca^2+^ simulations). RMSDs for the M1 (panel A), M2 (panel B), M3 (panel C), and M4 (panel D) regions from each subunit are shown. RMSDs were computed compared to the NMDAR crystal structure (calculated for C_α_s) for the last 100 ns (corresponding to 300–400 ns in Fig 6) following the targeted MD simulations (stage 4) (shown only for simulations with Ca^2+^ in the divalent cation binding site). Black, red, green and blue in each panel indicate subunits A, B, C and D, respectively. A and C correspond to the GluN1 subunits and B and D correspond to the GluN2A subunits.
(PNG)

S5 Fig GluN2A(S632) and GluN1(W608) distance measurements. GluN2A(S632) and GluN1(W608) distances are measured with Mg^2+^ (green line) and with Ca^2+^ (blue line) at the NMDAR divalent cation binding site during the last 100 ns of targeted MD simulations (stage 4).
(PNG)

S6 Fig Ligand coordination around Ca^2+^ during stage 4 model simulations. Oxygen ligands around Ca^2+^ at the NMDAR divalent cation binding site during the last 100 ns of targeted MD simulations (stage 4). OD1 and O are the atom names for oxygen in a side chain or backbone, respectively. The water molecules are shown in blue. Only water molecules with longest resident times are shown. Asn 61 and 373 correspond to GluN1 subunit N-sites; Asn 166 and 271 correspond to GluN2A subunit N-sites; Asn 167 and 272 correspond to GluN2A subunit N+1 sites.
(PNG)
