# Supplementary figures and images for: All atom NMDA receptor transmembrane domain model development and simulations in lipid bilayers and water
Source: PLoS One. 2017 Jun 5;12(6):e0177686. doi: 10.1371/journal.pone.0177686 (PMC5459333; doi:10.1371/journal.pone.0177686)

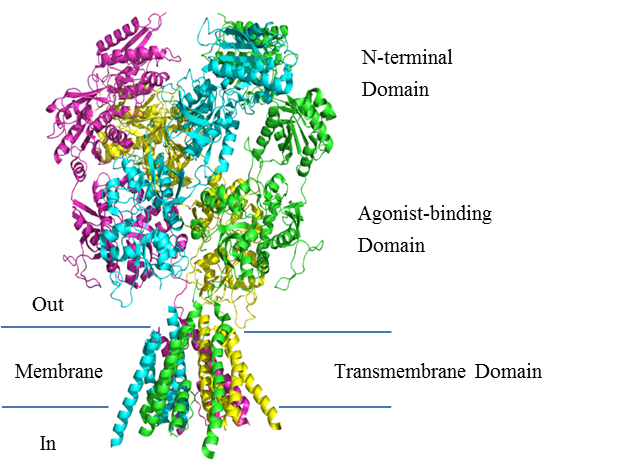

Supplement: S1 Fig — Domain organization of the NMDAR is shown for GluN1/2B subtype [16] (PDB ID: 4TLM). View of the receptor complex is parallel to the membrane, with the GluN1 subunits in cyan and yellow and the GluN2B subunits in green and purple. (PNG) [file pone.0177686.s001.png]

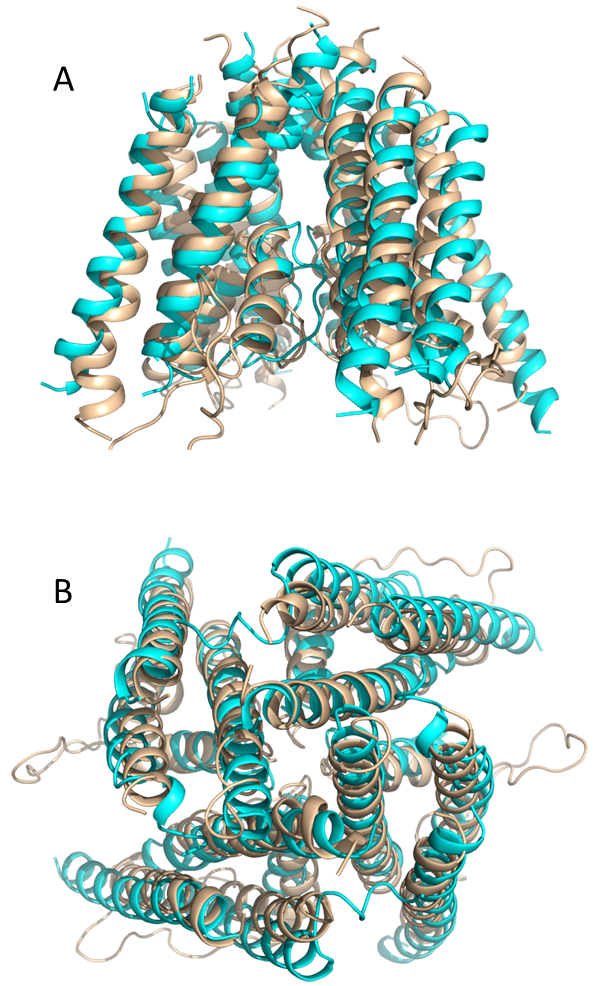

Supplement: S2 Fig — Superposition of the MD-optimized NMDAR TMD model (beige) and the Lee et al. [16]crystal structure (cyan). The total RMSD for all Cαs that were resolved in the crystal structure and included in the MD-optimized NMDAR TMD model is 3.79 Å. Panel A is side view and panel B is the top view (from the extracellular side) of the NMDAR. (PNG) [file pone.0177686.s002.png]

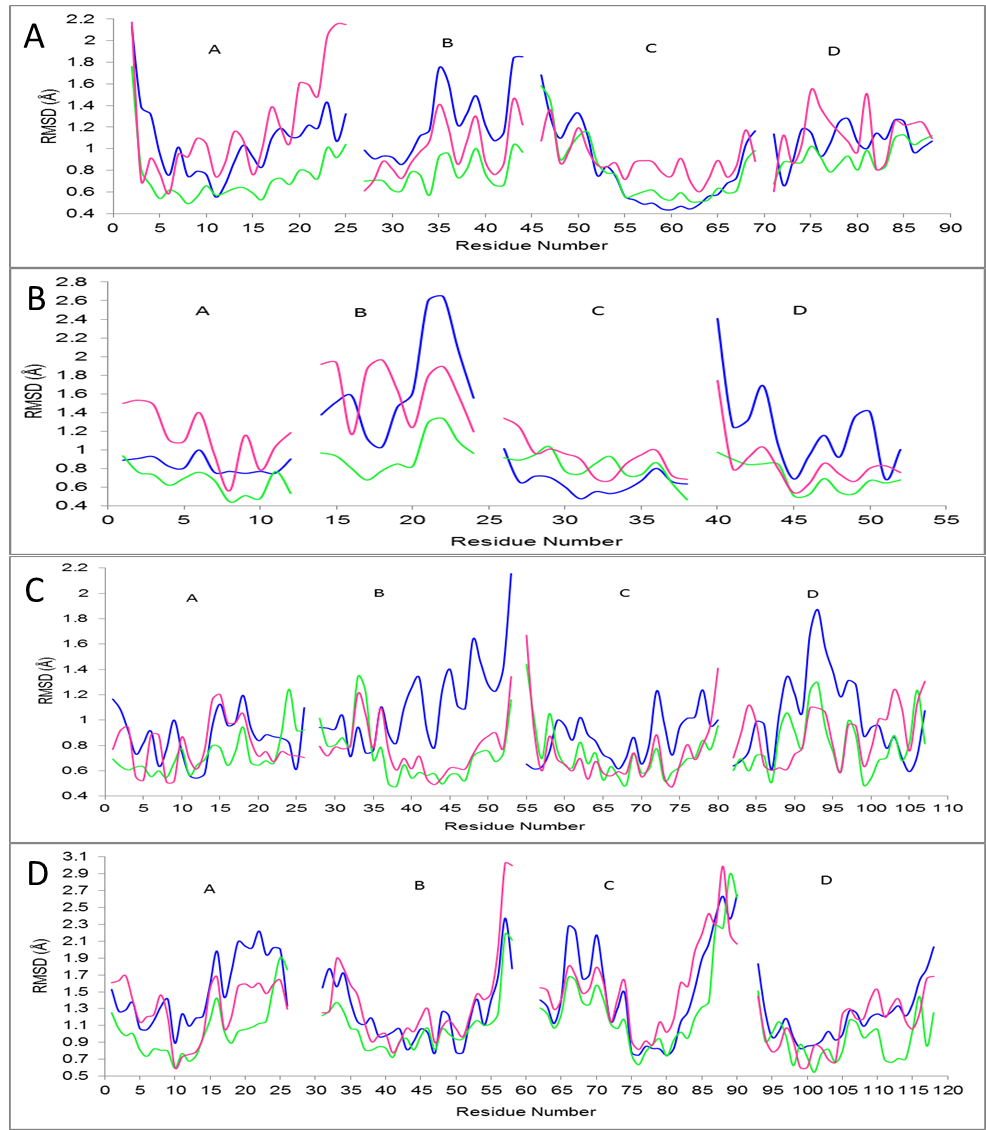

Supplement: S3 Fig — RMSDs were computed compared to the NMDAR crystal structure (calculated for Cαs) for the last 100 ns (corresponding to 300–400 ns in Fig 6) of the targeted MD simulations (stage 4). Simulations with Mg2+, Ca2+ and no ion in the divalent cation binding site are shown in green, blue and pink, respectively. In each panel, A and C correspond to GluN1 subunits and B and D correspond to GluN2A subunits. The numbering scheme is as follows: Panel A Residue numbers 2–25 and 65–88 correspond to GluN1 residues 558–581 in PDB ID: 4TLM. Residue numbers 27–44 and 46–63 correspond to GluN2A residues 551–568 in PDB ID: 4TLM. Panel B Residue numbers 1–13 and 26–38 correspond to GluN1 residues 592–604 in PDB ID: 4TLM. Residue numbers 14–24 and 40–50 correspond to GluN2A residues 586–596 in PDB ID: 4TLM. Panel C Residue numbers 1–26 and 82–107 correspond to GluN1 residues 617–642 in PDB ID: 4TLM. Residue numbers 28–53 and 55–80 correspond to GluN2A residues 611–636 in PDB ID: 4TLM. Panel D Residue numbers 1–27 and 31–58 correspond to GluN1 residues 800–827 in PDB ID: 4TLM. Residue numbers 62–89, and 91–118 correspond to GluN2A residues residue 800–827 in PDB ID: 4TLM. (PNG) [file pone.0177686.s003.png]

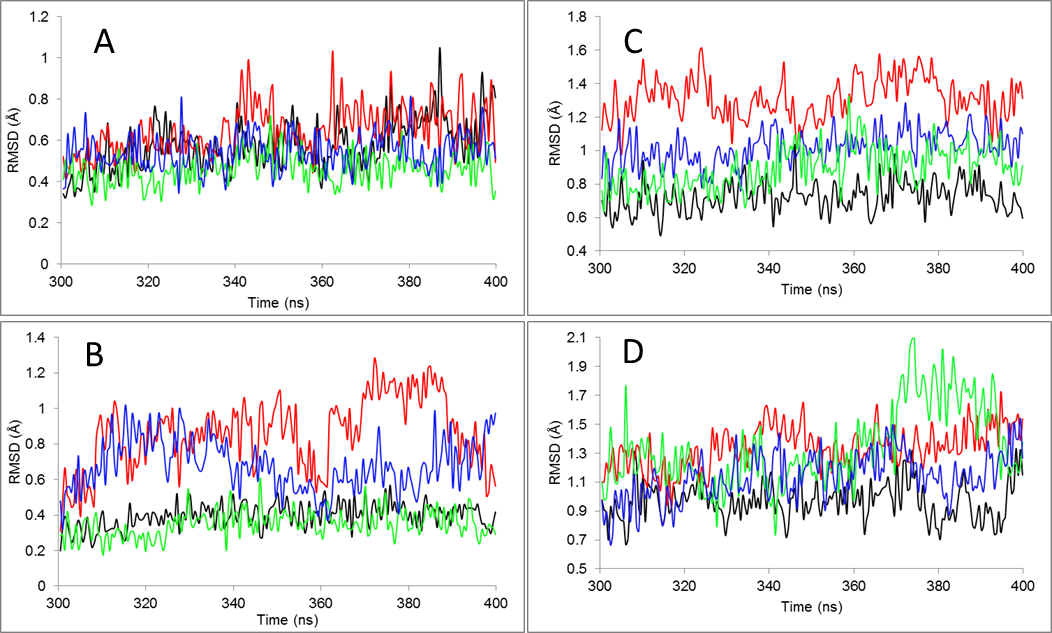

Supplement: S4 Fig — RMSDs for the M1 (panel A), M2 (panel B), M3 (panel C), and M4 (panel D) regions from each subunit are shown. RMSDs were computed compared to the NMDAR crystal structure (calculated for Cαs) for the last 100 ns (corresponding to 300–400 ns in Fig 6) following the targeted MD simulations (stage 4) (shown only for simulations with Ca2+ in the divalent cation binding site). Black, red, green and blue in each panel indicate subunits A, B, C and D, respectively. A and C correspond to the GluN1 subunits and B and D correspond to the GluN2A subunits. (PNG) [file pone.0177686.s004.png]

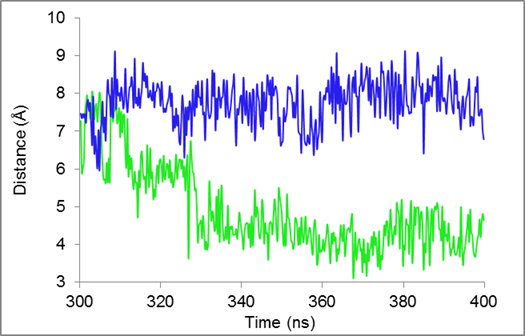

Supplement: S5 Fig — GluN2A(S632) and GluN1(W608) distances are measured with Mg2+ (green line) and with Ca2+ (blue line) at the NMDAR divalent cation binding site during the last 100 ns of targeted MD simulations (stage 4). (PNG) [file pone.0177686.s005.png]

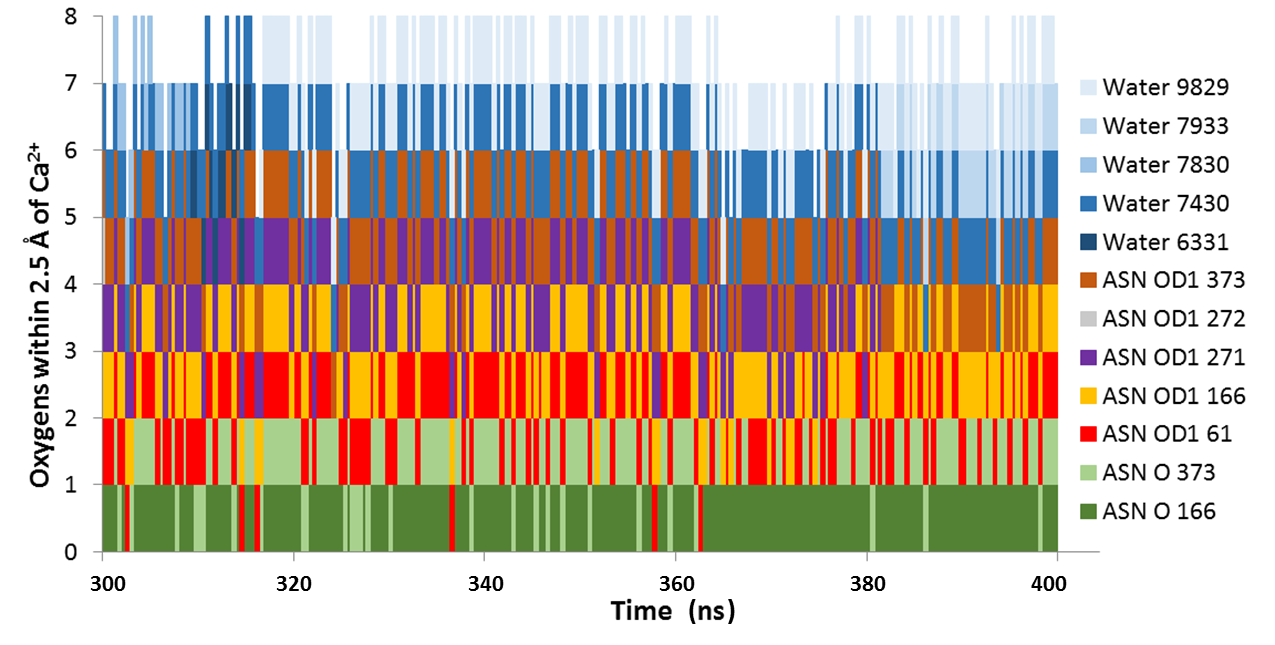

Supplement: S6 Fig — Oxygen ligands around Ca2+ at the NMDAR divalent cation binding site during the last 100 ns of targeted MD simulations (stage 4). OD1 and O are the atom names for oxygen in a side chain or backbone, respectively. The water molecules are shown in blue. Only water molecules with longest resident times are shown. Asn 61 and 373 correspond to GluN1 subunit N-sites; Asn 166 and 271 correspond to GluN2A subunit N-sites; Asn 167 and 272 correspond to GluN2A subunit N+1 sites. (PNG) [file pone.0177686.s006.png]
